# Clinico-pathological features of kidney disease in diabetic cases

**DOI:** 10.1007/s10157-018-1556-4

**Published:** 2018-03-21

**Authors:** Kengo Furuichi, Miho Shimizu, Hirokazu Okada, Ichiei Narita, Takashi Wada

**Affiliations:** 1 Kengo Furuichi, Division of Nephrology, Kanazawa University Hospital, 13-1 Takara-machi, Kanazawa, Ishikawa 920-8641 Japan; 2 Department of Nephrology, Faculty of Medicine, Saitama Medical University, 38 Morohongo, Moroyama-cho, Irumagun, Saitama 350-0451 Japan; 3 Division of Clinical Nephrology and Rheumatology, Kidney Research Center, Niigata University Graduate School of Medical and Dental Sciences, 1-757 Asahimachi-Dori, Chuo-ku, Niigata, 951-8510 Japan; 4 Department of Nephrology and Laboratory Medicine, Kanazawa University, 13-1 Takara-machi, Kanazawa, Ishikawa 920-8641 Japan

**Keywords:** Diabetic nephropathy, Diabetic kidney disease, Nephrosclerosis

## Abstract

Diabetic kidney disease is the major cause of end-stage kidney disease in developed countries. However, the onset of kidney disorder and the progression pattern of kidney dysfunction and proteinuria greatly vary cases by cases. Therefore, risk classification with clinical data and pathological findings is important. Recent clinico-pathological study with kidney biopsy samples from diabetic patients revealed that pathological changes of diabetic nephropathy are characteristic and have special impacts on prognosis in each clinical stage. Moreover, comparison of the clinico-pathological findings of diabetic nephropathy with hypertensive nephrosclerosis revealed that there are few differences in their pathological findings in cases with low albuminuria and preserved estimated glomerular filtration rate (eGFR). Because it is so difficult to clearly distinguish pure kidney lesions caused by diabetes and kidney lesions due to effects other than diabetes, it is vital that these overlapped pathological findings be confirmed on kidney biopsy in cases of early stage diabetes. Further research is warranted regarding the pathogenesis of diabetic nephropathy and indication of kidney biopsy in diabetic cases.

## Introduction

Diabetes causes various organ complications, with the kidney being one of the major target organs. Diabetic cases with kidney disease are the major cause of end-stage kidney disease in developed countries [1]. However, the onset and the progression pattern of kidney dysfunction and proteinuria greatly vary cases by cases [2–4]. Therefore, classification of the risks involved in the development and the progression of kidney disease is crucial. In addition, improvement in blood glucose control, the utilization of renin–angiotensin blockers, and an increase in the aging population changed the clinical manifestation of kidney disease in diabetic patients. Risk classification would also be beneficial for a therapeutic approach toward diabetic cases with kidney disease. Japanese classification of diabetic nephropathy and chronic kidney disease heat map are clinical classifications divided by albuminuria and estimated glomerular filtration rate (eGFR) [5 6, 7]. In addition to these clinical classification, pathological findings are important prognostic factors for kidney diseases, as well [8]. Moreover, kidney biopsy must be performed to exclude other kidney diseases besides diabetes. Recent reports on studies of kidney biopsies in diabetic cases revealed extremely variable changes of kidney diseases [9]. The incidence of retinopathy is more frequent in albuminuric cases than in normoalbuminuric cases. Moreover, cases with nodular lesions more frequently revealed evidence of diabetic retinopathy [10]. Therefore, it has been generally recognized that cases with diabetic nephropathy usually had diabetic retinopathy. However, our biopsy cohort revealed that 60% normoalbuminuria cases, 64% microalbuminuria cases, and 37% macroalbuminuria cases with diabetic nephropathy do not have diabetic retinopathy. Moreover, Japan diabetes complication study revealed that 65% of microalbuminuria cases with diabetic nephropathy do not have diabetic retinopathy [11]. Moreover, the meta-analysis with 26 papers revealed that the pooled sensitivity and specificity of diabetic retinopathy to predict diabetic nephropathy were 0.65 and 0.75, respectively [1, 12]. In addition, our biopsy cohort data revealed that diabetic nephropathy cases with retinopathy were poor prognosis of composite kidney events in Kaplan–Meier analysis (*p* < 0.05); and the hazard ratio is 1.8 in Cox analysis. These findings indicated that retinopathy is not always the indicator of nephropathy and the presence of retinopathy is poor prognostic factor for kidney function. Furthermore, recent autopsy study revealed that pathological changes of diabetic nephropathy developed before the onset of clinical findings (such as albuminuria and/or decreasing eGFR) [13–15]. Therefore, evaluation of pathological changes of kidney disease in diabetic cases would be clinically important. However, in general clinical practice, kidney biopsy is rarely performed in diabetic cases showing a typical diabetic clinical course of proteinuria and kidney dysfunction.

### Pathological findings of diabetic nephropathy

Diabetic nephropathy is usually diagnosed by clinical findings and kidney biopsy is performed in very limited cases; nevertheless, we collected and analyzed 600 kidney biopsy specimens of diabetic nephropathy [16, 17]. Diffuse lesions were observed in many cases of diabetic nephropathy even at the stage of normal albuminuria. Pathological findings such as nodular lesions and mesangiolysis were also observed in some cases at this stage. Cases with nodular lesions and mesangiolysis in the early stages had particularly poor prognosis of kidney disease. However, it is impossible to predict cases with the pathological findings using clinical parameters. Therefore, pathological evaluation is crucially important for clinical practice in kidney disease of diabetic patients.

In addition, interstitial lesions, such as interstitial cell infiltration and interstitial fibrosis or tubular atrophy, and vascular diseases, such as arteriolar hyalinosis and arteriosclerosis and vascular hyperplasia are also observed in biopsy specimens of the early stage diabetes cases. Moreover, recent study also reveals that arteriolar hyalinosis is a histological predictor for albuminuria increase and GFR decline in normo- and microalbuminuric cases with type 2 diabetes [18]. These findings also indicate that pathological evaluation is crucially important for clinical practice of diabetic cases.

### Pathological comparison of diabetic nephropathy with nephrosclerosis

**Fig. 1:**
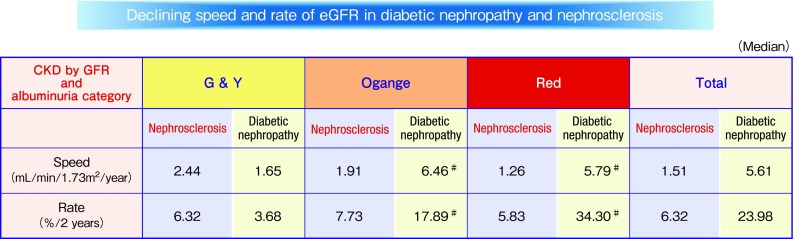
Declining speed and rate of eGFR in diabetic nepropathy and nephrosclerosis. *T* test. ^#^*p* < 0.05 to nephrosclerosis data from Ref. [16, 19]

**Fig. 2:**
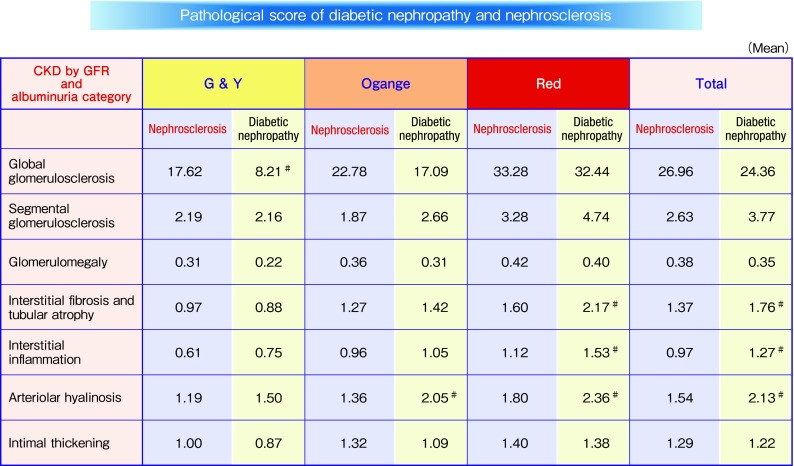
Pathological score of diabetic nephropathy and nephrosclerosis. *T* test. ^#^*p* < 0.05 to nephrosclerosis data from Ref. [16, 19]

It is well known; prognosis of kidney function in nephrosclerosis is better than that of diabetic nephropathy (Fig. 1). However, pathological comparison of diabetic nephropathy and nephrosclerosis was unclear. Nephrosclerosis is clinically diagnosed in cases with a history of hypertension and a small amount of proteinuria. Therefore, nephrosclerosis as well as diabetic nephropathy rarely performed kidney biopsy, and thus, the number of biopsy specimens is limited. A few reports on studies of kidney biopsy for nephrosclerosis have thus been reported. With the support of the Ministry of Health, Labour, and Welfare in Japan and the Japan Agency for Medical Research and Development, we collected and examined 184 kidney biopsies and clinical data on hypertensive nephrosclerosis throughout Japan [19]. Valuable findings were obtained by comparing the pathological findings of hypertensive nephrosclerosis and diabetic nephropathy. As mentioned in the previous section “Pathological findings of diabetic nephropathy”, interstitial lesions, such as interstitial cell infiltration and interstitial fibrosis or tubular atrophy, and vascular diseases such as arteriolar hyalinosis and arteriosclerosis were observed even in the early stage of diabetic nephropathy. The presence of these lesions in diabetic nephropathy is similar to that in nephrosclerosis. We compared the pathological findings of diabetic nephropathy with hypertensive nephrosclerosis, and found no difference in their pathological findings in cases with low albuminuria and preserved eGFR (Fig. 2). These findings indicate that hypertension may affect pathological changes in cases with low albuminuria and preserved eGFR in diabetic nephropathy. In contrast to these interstitial and vascular lesions, the rate of global nephrosclerosis is significantly high in patients with nephrosclerosis in Green and Yellow category. Body mass index (BMI), and systolic and diastolic blood pressure were higher in patients with nephrosclerosis than diabetic nephropathy in Green and Yellow category (data not shown). Therefore, we speculate that this finding indicates that BMI and high blood pressure would affect on pathogenesis of global nephrosclerosis in Green and Yellow category. Further kidney biopsy studies are required to confirm these speculations.

### Pathological changes of kidney biopsy specimens from diabetic cases

Diabetic kidney disease (DKD) usually refers to cases of clinically diagnosed kidney disease with diabetes. However, kidney biopsy and histological exclusion of primary glomerulonephritis are important in the treatment of DKD. A recent study of 129 biopsy-proven cases (59% were nephrotic-range proteinuria) revealed that 63% of cases were diabetic nephropathy and 37% were non-diabetic kidney disease (membranous nephropathy, 42%; IgA nephropathy, 15%; and minimal change disease, 10%) [20]. Moreover, a review article on kidney biopsy for diabetic cases reported that the prevalence of diabetic nephropathy was extremely variable reports by reports, and ranged from 7 to 94% of the overall cases, as was that of non-diabetic kidney disease (3–83%) and mixed forms (4–46%). Non-diabetic kidney disease included IgA nephropathy (3–59%), membranous nephropathy (7–35%), focal-segmental glomerulosclerosis (17–38%), and acute interstitial nephritis (18–49%) ^9^, indicating the importance of kidney biopsies in cases of clinically diagnosed DKD.

Conversely, in type 2 diabetic cases with chronic glomerulonephritis, hyperglycemia and metabolic disorder have an effect on primary glomerular lesions and vascular lesions in kidney disease progression. In addition to diabetes, various factors such as blood pressure, smoking, obesity, as well as genetic background may also affect the pathological changes of primary glomerular or tubulointerstitial disease [21]. Moreover, a lot of studies are also being conducted on the influence of obesity on diabetic changes in kidney disease. At the present time, it is unclear that obesity affects on diabetic changes in kidney disease directly or indirectly. Anyway, various factors are involved in the formation of kidney lesions in type 2 diabetes. Therefore, it becomes difficult to clearly distinguish pure kidney lesions caused by diabetes and kidney lesions caused by effects other than diabetes. However, it is important to diagnose and treat kidney disease by discriminating between primary kidney disease with diabetes mellitus and diabetic nephropathy, and it is necessary to perform kidney biopsy to exclude other kidney diseases from diabetic nephropathy.

Timing to perform the kidney biopsy in diabetic cases is a very important issue. Although the pathological evaluation of kidney biopsy specimens for diabetic nephropathy has many beneficial values, there is no clear indication for kidney biopsy so far. Especially in case with normoalbuminuria or microalbuminuria, pathological findings’ assessment is clinically valuable [16, 17]. However, kidney biopsy has some risks of bleeding, infection, and so on. Therefore, kidney biopsy for diabetic case could be performed under the informed consent of risk and benefit of the biopsy so far. Cases with atypical clinical course, such as rapid increase of albuminuria and progressive eGFR decline, would be candidates of kidney biopsy. It should be important to accumulate the biopsy cases of diabetic case and to verify the findings and prognosis. Under the accumulating knowledge of kidney biopsy value for diabetic cases, additional discussion will be necessary to reach a consensus of kidney biopsy indication for diabetic cases.

### Kidney disease in diabetic cases

**Fig. 3:**
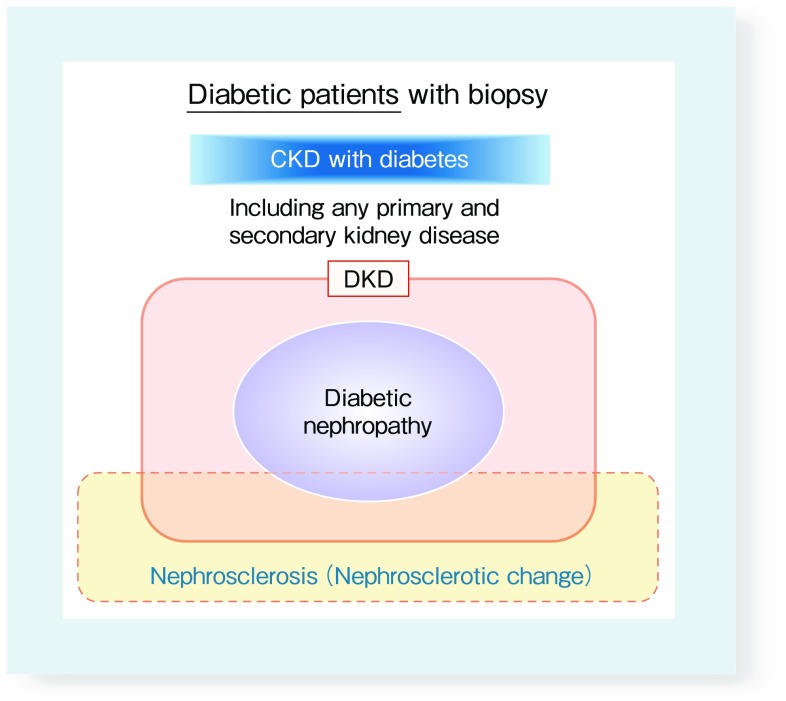
A Venn diagram of the notions of diabetic kidney disease and diabetic nephropathy

Diabetes affect in pathogenesis of primary or other secondary kidney disease in various degrees. For pathogenesis of kidney disease, it would be difficult to clearly distinguish the effects of diabetes with other primary or other secondary kidney disease. Therefore, we have to use several terms differently. From the viewpoints of kidney biopsy for diabetic cases, “DKD” is kidney specimens for typical diabetic changes at least in part. It may include primary or secondary kidney disease. On the other hand, “diabetic nephropathy” is kidney specimens with mainly typical diabetic change. It should be excluded primary or other secondary kidney diseases. In contrast to those terms, “CKD with diabetes” is kidney biopsy specimens from diabetic cases with any primary and secondary kidney diseases (Fig. 3). These concepts are unsettled, and the definition are unclear in some part so far. Further discussion is required in this point in future.

**Fig. 4:**
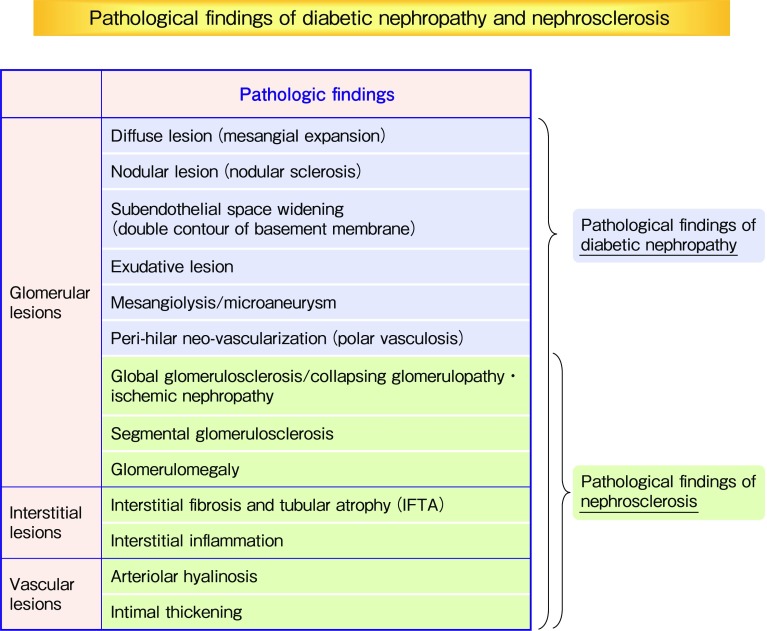
Pathological findings of diabetic nephropathy and nephrosclerosis

Nephrosclerotic lesions, such as glomerular sclerosis and arteriosclerosis, can be also found in many kidney biopsy sediments. However, it is difficult to clearly define the specific pathological changes of nephrosclerosis. The pathological changes of nephrosclerosis are often included in the pathological changes of diabetic nephropathy. In the pathological diagnostic manual for diabetic nephropathy and nephrosclerosis in Japan published in 2014, pathological findings of nephrosclerosis are included in the pathological findings of diabetic nephropathy (Fig. 4).

Although much discussion is required on this issue, the distinction between biopsy-proven diabetic nephropathy and primary kidney disease with diabetes is clinically important. Moreover, further clinical and basic research about kidney changes in diabetic cases is required.
